# Assessment of composite motif discovery methods

**DOI:** 10.1186/1471-2105-9-123

**Published:** 2008-02-26

**Authors:** Kjetil Klepper, Geir K Sandve, Osman Abul, Jostein Johansen, Finn Drablos

**Affiliations:** 1 Department of Cancer Reasearch and Molecular Medicine, Norwegian University of Science and Technology, Trondheim, Norway; 2 Department of Computer and Information Science, Norwegian University of Science and Technology, Trondheim, Norway; 3 Department of Computer Engineering, TOBB University of Economics and Technology, Ankara, Turkey

## Abstract

**Background:**

Computational discovery of regulatory elements is an important area of bioinformatics research and more than a hundred motif discovery methods have been published. Traditionally, most of these methods have addressed the problem of *single motif discovery *– discovering binding motifs for individual transcription factors. In higher organisms, however, transcription factors usually act in combination with nearby bound factors to induce specific regulatory behaviours. Hence, recent focus has shifted from single motifs to the discovery of sets of motifs bound by multiple cooperating transcription factors, so called *composite motifs *or *cis-regulatory modules*. Given the large number and diversity of methods available, independent assessment of methods becomes important. Although there have been several benchmark studies of single motif discovery, no similar studies have previously been conducted concerning composite motif discovery.

**Results:**

We have developed a benchmarking framework for composite motif discovery and used it to evaluate the performance of eight published module discovery tools. Benchmark datasets were constructed based on real genomic sequences containing experimentally verified regulatory modules, and the module discovery programs were asked to predict both the locations of these modules and to specify the single motifs involved. To aid the programs in their search, we provided position weight matrices corresponding to the binding motifs of the transcription factors involved. In addition, selections of decoy matrices were mixed with the genuine matrices on one dataset to test the response of programs to varying levels of noise.

**Conclusion:**

Although some of the methods tested tended to score somewhat better than others overall, there were still large variations between individual datasets and no single method performed consistently better than the rest in all situations. The variation in performance on individual datasets also shows that the new benchmark datasets represents a suitable variety of challenges to most methods for module discovery.

## Background

A key step in the process of gene regulation is the binding of transcription factors to specific *cis*-regulatory regions of the genome, usually located in the proximal promoter upstream of target genes or in distal enhancer regions [1,2]. Each transcription factor recognizes and binds to a more or less distinct nucleotide pattern – a *motif *– thereby regulating the expression of the nearby gene. Determining the location and specificity of each transcription factor binding site in the genome is thus an important prerequisite for reconstructing the gene regulatory network of an organism.

Since establishing these binding sites experimentally is a rather laborious process, much effort has been made to develop methods that can automatically discover such binding sites and motifs directly from genomic sequence data. More than a hundred methods have already been proposed [3], and new methods are published nearly every month. There is a large diversity in the algorithms and models used, and the field has not yet reached agreement on the optimal approach. Most methods search for short, statistically overrepresented patterns in a set of sequences believed to be enriched in binding sites for particular transcription factors, such as promoter sequences from coregulated genes or orthologous genes in distantly related species.

In higher organism, however, transcription factors seldom function in isolation, but act in concert with nearby bound factors in a combinatorial manner to induce specific regulatory behaviours. A set of binding motifs associated with a cooperating set of transcription factors is called a *composite motif *or *cis-regulatory module*. In recent years, the field of computational motif discovery has therefore shifted from the detection of single motifs towards the discovery of entire regulatory modules.

The diversity of approaches to module discovery is even greater than for single motif discovery, and methods vary widely in what they expect as input and what they provide as output. For instance, methods like Co-Bind [4], LOGOS [5] and CisModule [6] expect only a set of coregulated or orthologous promoter sequences as input and are able to infer both the location and the structure of modules with few prior assumptions regarding their nature. These programs infer an internal model that includes a representation of each individual transcription factor binding motif as well as constraints on the distances between them. On the other hand, programs such as LRA [7] and Hexdiff [8] demand as input a collection of already known module sites to serve as training data. The known positive sites are used along with negative sequence examples to build a model representation which can then be compared to new sequences in order to identify novel module instances. Searching for new matches to a previously defined model might be considered a special case of module discovery and is often referred to as module *scanning*. Programs that specialize in searching for modules this way without inferring the models themselves include ModuleInspector [9] and ModuleScanner [10]. The general problem of module discovery, however, usually involves inferring both a model representation of the modules and to find their locations in the sequences.

Most module discovery methods require users to supply a set of candidate single motif models in the form of IUPAC consensus strings or position weight matrices (PWM) [11]. These are used to discover putative transcription factor binding sites in the sequences, and the programs then search for significant combinations of such binding sites to report as modules.

What constitutes a significant combination varies between methods. MSCAN [12], for instance, searches for regions within sequences that have unusually high densities of binding sites, more so than would be expected from chance alone. The types of the binding motifs are irrelevant, however, and each potential module instance is analyzed independently from the rest. Other tools, like ModuleSearcher [10], Composite Module Analyst [13] and CREME [14], search for specific combinations of motifs that co-occur multiple times in regulatory regions of related genes.

With an increasing number of programs available, both for single and composite motif discovery, there is a growing need among end users for reliable and unbiased information regarding the comparative merits of different approaches. A few independent investigations have been undertaken to assess the performance of selected single motif discovery methods, for instance by Sze *et al*. [15] and Hu *et al*. [16]. The most comprehensive benchmark study to date was carried out by Tompa *et al*. and included thirteen of the most popular single motif discovery methods [17]. The authors of this study also provided a web service to enable new methods to be assessed and compared to the original methods using the same datasets.

However, in spite of the increased interest in regulatory modules, we are not aware of any similar independent benchmarking efforts that have been undertaken with respect to composite motif discovery.

## Results

We have developed a framework for assessing and comparing the performance of methods for the discovery of composite motifs. Sequence sets containing real, experimentally verified modules are made available for download through our web service, and users can test programs of their own choice on these datasets and submit the results back to the web service to get the predictions evaluated. Results are presented both as tabulated values and in graphical format, and performances of different methods can be compared. Since most module discovery tools require users to input candidate motifs, each sequence dataset is supplemented by a set of PWMs capable of detecting the binding sites involved in the modules. To test how programs respond to varying levels of noise in the PWM sets, we created extended PWM sets for one of our datasets where the genuine matrices were mixed with various decoy matrices.

### Scoring predictions

We adopted a simple and general definition of a module: a *module *is a *cis*-regulatory element consisting of a collection of single binding sites for transcription factors. A module is thus characterized by only two aspects in our framework: its *location *in a sequence and its *composition*, that is, the set of transcription factor binding motifs involved. A module's location is further defined as the smallest contiguous sequence segment encompassing all the single binding sites in the module, including also the intervening bases. For our purpose, the composition of a module is represented by a set of PWM identifiers. Different modules that share the same composition are said to belong to the same *module class*. Module class definitions may also be limited by structural *constraints*. These are rules governing, among others, the strand bias, order and distances between the transcription factor binding sites of modules of the same class. Since it requires a substantial effort to determine these constraints experimentally, this kind of information is available for a very limited number of classes. Few methods also report such module constraints explicitly. Consequently, we have chosen not to consider this aspect of modules further in our framework, at least for the time being.

Module discovery programs are requested to predict both the location of modules and to identify the motifs involved by naming the proper PWMs. However, not all programs are able to perform both these tasks. The MCAST program [18], for instance, only reports the location of predicted modules, even though it uses a set of PWMs to detect single binding sites internally. On the other hand, programs that discover single motifs *de novo *without relying on pre-constructed matrices have, of course, no way of correctly naming the motifs involved. Methods like that of Perco *et al*. [19] and GCMD [20] identify modules by looking for groups of PWMs whose binding sites consistently appear together in multiple sequences, but disregard any further information about the precise position of these sites. Hence, such programs only report the composition of modules but not their location. By assessing the location and composition aspects of modules separately, our framework can equally well be used with programs that predict only one or the other.

To measure prediction accuracy of methods with respect to module location, we have used the *nucleotide-level correlation coefficient *(*nCC*). This statistic has been widely used before, among others, for coding region identification and gene structure prediction [21]. It was also adopted by Tompa *et al*. to evaluate binding site predictions in their single motif discovery benchmark study. The value of *nCC *lies in the range -1 to +1. A score of +1 indicates that a prediction is coincident with the correct answer; whereas a score of -1 means that the prediction is exactly the inverse of the correct answer. Random predictions will generally result in *nCC*-values close to zero.

$$nCC=\frac{TP\cdot TN-FN\cdot FP}{\sqrt{\left(TP+FN)(TN+FP)(TP+FP)(TN+FN\right)}}$$

Here, *TP *is the number of nucleotides in a sequence that are correctly predicted by a program as belonging to a module, while *TN *is the number of nucleotides correctly identified as background. *FN *is the number of true module nucleotides incorrectly classified as background, and *FP *is the number of background nucleotides incorrectly classified as belonging to a module.

A similar statistic, the *motif-level correlation coefficient *(*mCC*), was used to evaluate prediction accuracy with respect to module composition. The definition of *mCC *follows that of *nCC*, except that instead of counting the number of nucleotides, we count the number of single motifs (or PWMs) correctly or incorrectly classified as being part of a module or not. Hence, for *mCC*, *TP *is the number of PWMs correctly identified as constituents of the module, while *FP *is the number of PWMs incorrectly predicted as being part of a module. Note that the correlation statistics, as defined here, are only applicable when both the datasets and the predictions made by a program contain a combination of module and non-module instances, if not, the divisor will be zero and the value of the statistic will be undefined. Consequently, the *mCC*-score is only informative when the set of PWMs supplied to a module discovery program contains false positives, i.e. additional matrices besides those that are actually involved in the modules. Final scores for each dataset are obtained by summing up *TP*, *FP*, *TN *and *FN *over all sequences before calculating the correlation scores. If no module predictions are made on a set of sequences, the resulting scores for *nCC *and *mCC *are assigned a value of zero rather than being left undefined. In addition to *CC *scores, several other statistics mentioned in [17] such as *sensitivity*, *specificity*, *positive predictive value*, *performance coefficient *(phi-score) and *average site performance *are calculated for both nucleotide- and motif-level.

### Datasets

We compiled three datasets from sequences containing experimentally verified regulatory modules. The first and the last two datasets have different characteristics and were chosen to complement each other to test methods under different conditions.

Our main dataset was based on annotated composite motifs from the TRANSCompel database [22]. The modules selected for this dataset are small, each consisting of exactly two single binding sites for different transcription factors (TFs), but we specifically chose modules that had multiple similar instances in several sequences. Sequences containing modules from the same class were grouped together producing ten sequence sets named after their constituent single motifs as shown in Table 1. Each of the sequences in a set contained at least one copy of the module with the same two motifs, but the order, orientation and distance between the TFBS could vary between sequences. Separate PWM collections, with matrices for the two single motifs involved, were constructed for each of the sequence sets. All in all there were eleven distinct single TF binding motifs in our full TRANSCompel dataset, and PWMs representing these motifs were collected from the companion TRANSFAC database [22]. Since TRANSFAC often contains several different PWMs for each motif, we grouped all the matrices corresponding to a particular motif into an equivalence set, essentially treating these PWMs as if they were one and the same with respect to prediction and scoring. In addition to the TRANSFAC matrix sets, we also constructed eleven custom matrices that were specifically tailored to the particular motifs and binding sites present in the sequences (see Methods). Assessment of module discovery programs on the TRANSCompel dataset was conducted using both the TRANSFAC sets and the customized PWM sets independently. The motivation for using two different PWM sets was to test the stability of methods and examine how the specific representations used for single motifs might influence the ability of methods to find the correct modules.

**Table 1:** Datasets

**Sequence set**	**Sequences**	**Modules**	**Total size (bp)**	**Module size, min-max (avg)**
AP1-Ets	16	17	14860	14 – 99 (27)
AP1-NFAT	8	11	6893	14 – 19 (16)
AP1-NFκB	7	8	6532	18 – 135 (53)
CEBP-NFκB	8	8	7308	44 – 118 (84)
Ebox-Ets	4	6	3489	16 – 50 (25)
Ets-AML	5	5	4053	13 – 30 (19)
IRF-NFκB	6	6	5344	23 – 71 (43)
NFκB-HMGIY	6	7	5393	10 – 32 (13)
PU1-IRF	5	5	4530	12 – 14 (13)
Sp1-Ets	7	8	5787	16 – 117 (37)
**Liver**	12	14	11943	26 – 176 (112)
**Muscle**	24	24	20427	14 – 294 (120)

A brief overview of the ten TRANSCompel sequence sets and the liver and muscle datasets used in the assessment. Further information can be found in Additional File 1.

The two last datasets were based on combinations of TFBS found in the regulatory regions of genes specifically expressed in liver [23] and muscle [7] cells. The modules here are usually larger compared to the TRANSCompel modules, containing up to nine binding sites for four different motifs in the liver regulatory regions and up to eight sites for five motifs in the muscle regions. PWMs for these motifs were taken from the respective publications. The composition of the modules in these two datasets is variable; modules can contain multiple binding sites for the same motifs and not all motifs are present in every module.

While most programs require candidate PWMs to be entered, this can pose a problem for users who might not always know in advance the kind of modules that should be present in a sequence or which transcription factors that might bind. It could be the case, for instance, that a researcher has only a set of promoters from a coregulated set of genes and is interested in identifying the hitherto unknown module that controls the common expression of these genes. A popular strategy then is to employ an excessive set of PWMs which, hopefully, also includes the appropriate matrices. An extreme, but not unlikely, scenario would be to use all the matrices available from a published compilation like TRANSFAC (774 matrices in release 9.4) or Jaspar [24] (123 core matrices). Although this approach will inevitably lead to lots of false positive PWM matches that might thwart the module discovery process, good module discovery tools should nonetheless be able to report the true module instances without simultaneously predicting too many spurious occurrences.

To simulate these conditions and test methods' response to noisy PWM sets, each PWM set under the TRANSCompel dataset was issued in multiple versions with progressively more decoy matrices added to the set of true annotated motifs. Decoy matrices were randomly sampled from the complete TRANSFAC compilation after removing the matrices corresponding to the true motifs for a sequence set. Decoy sets are available at 50%, 75%, 90%, 95% and 99% levels, where the percentage number relates the amount of decoy matrices in the set. Thus, a custom PWM set at the 90% level includes 2 genuine matrices and 18 decoy matrices. The number of decoy matrices in the TRANSFAC PWM sets varies with each module class but is always higher than for the custom sets at the same percentage level. Information on the exact number of PWMs in each set is available in Additional File 1. The 99% sets include as decoys all of the matrices from TRANSFAC which do not correspond to the correct motifs. They are called "99%" for consistency, although the actual percentage of decoys ranges between 95% and 99% depending on the module class. To avert artefacts stemming from possibly biased selections of decoys, all decoy sets (except at the 99% level) consist of ten independently sampled decoy collections, and the final correlation statistics for a decoy level are calculated by averaging prediction scores made from using each collection in turn. This also means that variation due to any stochastic nature of algorithms will be averaged over ten independent runs.

### Benchmark of module discovery methods

Using our assessment framework, we benchmarked eight published methods for module discovery: *CisModule *[6], *Cister *[25], *Cluster-Buster *[26], *Composite Module Analyst (CMA) *[13], *MCAST *[18], *ModuleSearcher *[10], *MSCAN *[12] and *Stubb *[27]. See Table 2 for brief descriptions of each of these methods. CisModule, CMA and ModuleSearcher process all the sequences in a dataset simultaneously and look for instances of similar modules across multiple sequences. The other methods examine the sequences individually, although Stubb considers multiple instances of similar modules within the same sequence. Except for MCAST, which does not report module composition, all the programs report both the location and composition of modules. CisModule, however, predicts modules *de novo *without relying on supplied PWM sets and so does not name the single motifs involved the way we require. Hence, motif-level scores were not calculated for MCAST and CisModule. Cluster-Buster and MCAST report the full module segments, while the rest of the methods list the positions of the PWM hits in the modules. In these cases we extracted the start position of the first reported binding site and the end position of the last binding site and used these as the boundaries of a module prediction.

**Table 2:** Description of module discovery tools

CisModule	CisModule models the structure of sequences with a two-level hierarchical mixture-model and uses a Bayesian approach with Gibbs sampling to simultaneously infer the modules, TFBSs and PWMs based on their joint posterior distribution, which is the probability of a model given the input sequence set. At the first level, sequences are viewed as a mixture of module instances and background. At the second level, modules are modelled as a mixture of motifs and inter-module background. Parameters of the model include the widths and representations (PWMs) of single motifs and parameters related to distances between modules and between TFBS within modules. From a random initialization, CisModule iteratively cycles through steps of parameter update and module-motif detection. New parameter values are sampled from their conditional posterior distributions based on the currently predicted modules and motifs, and new predictions of modules and TFBSs are then sampled based on these updated parameter values. Positions in the sequences where the marginal posterior probability of being sampled within modules was greater than 0.5 were output as module predictions.
Cister	Given a set of PWMs and parameters specifying the expected number of motifs in modules, the expected distances between motifs in modules and the expected distance between modules, *Cister *builds a Hidden Markov Model (HMM) with three basic states: *motif*, *intra-module background *and *inter-module background*. Transition probabilities between these states follow geometric distributions according to the expected values input by the user. In the motif state, one of the PWMs is chosen uniformly at random and used to decide the probabilities of outputting nucleotides. Background-state emission probabilities are estimated from a sliding window centered on the current base in the query sequence. From this HMM, the posterior probability that each base in the input sequence was generated from a module state as opposed to the inter-module state can be calculated. Predicted modules are defined to occur at local maxima of this posterior probability curve where the value is at least 0.5 and no larger value is observed within 1200 bp.
Cluster-Buster	Cluster-Buster is developed by the same group that made *Cister *and is designed to search for clusters of pre-specified motifs in nucleotide sequences. Like Cister, Cluster-Buster constructs a HMM-model based on the user-supplied PWMs, an expected distance between motifs in clusters and background distributions estimated from the input sequence over sliding windows. Log likelihood ratios are used to determine whether a sequence is more likely to be generated by a "cluster-model" or a "background-model". Cluster-Buster uses a linear time heuristic to rapidly estimate log likelihood ratios for all subsequences of the input sequence and outputs those subsequences with ratios above a specified threshold that do not overlap with other higher scoring subsequences.
Composite Module Analyst (CMA)	The promoter model in CMA is expressed as a Boolean combination of one or more *composite modules *(CM), each of which consist of a set of single, independent motifs as well as pairs of motifs that must obey certain constraints on distance and orientation. Given a candidate promoter model, the method searches for potential matches to the CMs in the sequences, and a final promoter score is calculated after the presence or absence of each CM is established. CMA employs a Genetic Algorithm to search for the promoter model which best discriminates between a set of positive (co-regulated) and a set of negative sequences. The fitness function is based on a linear combination of several properties of the distribution of the promoter scores and of the individual CM scores in the two sequence sets.
MCAST	MCAST builds a HMM-model consisting of an intra-module state, an inter-module state and motif-states based on the supplied PWMs. The score for a motif-state is called a *p*-score and is the negative logarithm of the *p*-value of a log-odds score based on the probability of a segment in the target sequence being generated either by the PWM or a fixed, user-specified zero-order Markov background model. MCAST forbids transitions into motif-states that result in *p*-scores lower than some chosen threshold. Some state transitions are associated with certain costs. For instance, entering the inter-module state from a motif-state incurs a large one-time penalty while cycling through the intra-module state incurs smaller penalties for each nucleotide emitted. The Viterbi algorithm is used to find the highest scoring path through the HMM with respect to the input sequence, classifying each position in the sequence as either belonging to a module or to the background. Potential module segments are scored according to the number of motifs in the module and the *p*-scores of these motifs and are penalized by the number of intra-module background bases. Finally, modules are ranked according to the estimated *E*-values of these scores.
ModuleSearcher	Given a list of PWM hits with match scores for putative TFBSs in a sequence set, ModuleSearcher finds the module model (set of *k *PWMs) that best fits the sequences. The score of a module model is calculated as the sum of scores over all sequences, and the score function for a single sequence is based on the best scoring set of TFBSs in the sequence that corresponds to the PWMs in the module model. To be considered a valid TFBS set the binding sites must all lie within a short window, and the user can choose to ignore TFBS sets with overlapping binding sites or penalize sets that lack sites for some PWMs. An A*-algorithm (or alternatively a Genetic Algorithm) is employed to search the space of possible subsets of *k *motifs from the full PWM library in order to find the highest scoring module model.
MSCAN	MSCAN discovers modules by evaluating the combined statistical significance of sets of potential non-overlapping TF binding sites in a sliding window along the input sequence. PWMs are compared against each position within the window to obtain match scores, and *p*-values are calculated as the probability of obtaining similar or higher scores at a specific position in a random sequence with nucleotide distribution similar to the distribution in the window. MSCAN proceeds by calculating significance scores for all combinations of up to *k *binding sites in the window and then selects the optimal combination (the one with the lowest score). A prediction is output if a final *p*-value computed from this score is less than some user-specified threshold.
Stubb	The HMM used by Stubb consists of motif states based on supplied PWMs and a single background state based on a *k*th-order Markov model with probability distribution estimated from a sliding window. The scoring function is the log likelihood ratio that the sequence within a limited window was more likely generated by the full model than with a HMM consisting of only the background state. Unlike the other HMM methods presented here, the transition probabilities between states in Stubb are not based on user-input expectancies, but are estimated from the sequence using the Baum-Welch algorithm. This procedure finds the set of transition probabilities that maximizes the scoring function. If Stubb finds that some motifs are highly correlated with respect to order, it can make use of *correlated transition probabilities*. This means that the probability of entering a specific motif state will dependent on which previous motif was output. Stubb can also utilize phylogenetic comparisons between sequences from multiple species to highlight potentially regulatory modules.

The table contains short descriptions of the eight methods included in the assessment. All methods except for CisModule rely on supplied PWMs and consider matches on both strands, usually with equal probability (however, Stubb can estimate strand biases for all PWMs in a preprocessing step). Not all methods are able to consider overlapping single binding sites, which do occur in a few modules.

We generally relied on default parameter settings for all programs. However, since choosing the proper parameter values can sometimes prove crucial for a method's performance, we decided to provide the programs with a few general clues where applicable; specifically, that the size of modules should not exceed 200 bp (300 bp in the muscle dataset) and that the modules should consist of exactly two single binding sites for different TFs in the TRANSCompel dataset but possibly up to ten binding sites for four and five different TFs on the liver and muscle sets respectively. Furthermore, binding sites could potentially overlap and the composition of the modules in liver and muscle sets should be allowed to vary between sequences.

**Figure 1:**
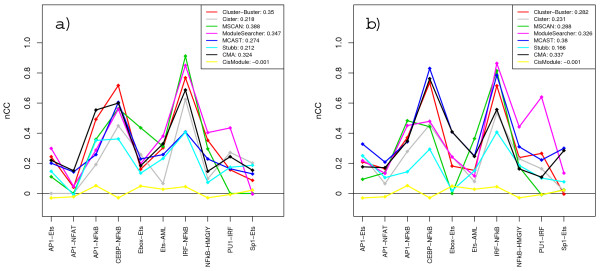
**Nucleotide-level correlation scores on the TRANSCompel dataset**. The graphs show *nCC *scores for each of the ten sequence sets in the TRANSCompel dataset when methods are supplied with TRANSFAC PWM sets (a) and custom matrices (b).

**Figure 2:**
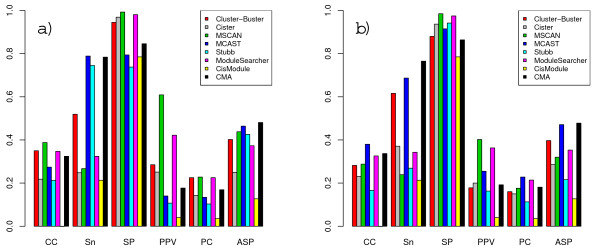
**Combined performance scores on the full TRANSCompel dataset**. Combined nucleotide-level scores obtained for different performance measures when using TRANSFAC PWM sets (a) and custom matrices (b).

Figures 1a and 1b show the resulting nucleotide-level correlation scores on each sequence set in the TRANSCompel dataset when methods were supplied with TRANSFAC matrices and custom matrices respectively. The scores vary widely between individual sequence sets but are generally fairly well correlated between methods, so that most methods tend to get high (or low) scores on the same sets. The notable exception is CisModule which performs poorly on all sequence sets. The correlation suggests that some sequence sets are inherently more easy (or difficult) to tackle than others. Scores for CEBP-NFκB and IRF-NFκB are the highest overall. The reasons why these sets are generally easy to predict might be that their modules are quite long and the matrices representing the single binding motifs have high information content (see Table 3 and Additional File 1). Conversely, the short size of the modules and the low information content of PWMs for AP1-NFAT would make this a hard sequence set. We also calculated combined scores for the whole TRANSCompel dataset which are shown in the inset legends of Figure 1 and graphically in Figure 2. These combined scores were obtained by summing up TP, TN, FP, FN over all sequence sets when calculating the score measures. The highest combined *nCC *scores achieved were 0.388 with the TRANSFAC matrices (MSCAN) and 0.38 with custom matrices (MCAST). The average performances across all methods were also about the same with the two PWM sets. Some methods performed quite differently depending on the PWMs, however. For instance, MCAST scored much better using custom matrices than with TRANSFAC matrices, while MSCAN and Cluster-Buster did a better with job with TRANSFAC. The rank order of methods is thus somewhat altered between the two cases. Still, some tendencies remain: CMA, Cluster-Buster, MCAST, ModuleSearcher and MSCAN occupy the top five positions in both cases, followed by Cister and Stubb and then finally CisModule which consistently scored lowest.

**Table 3:** Correlations between dataset properties and *nCC *scores

	**TRANSFAC PWMs**		**Custom PWMs**	
	
	Average *nCC*	Highest *nCC*	Average *nCC*	Highest *nCC*
Number of sequences	-0.23	-0.16	-0.23	-0.05
Length of shortest sequence	0.30	0.18	0.30	0.13
Average sequence length	0.40	0.33	0.42	0.43
Total sequence set length	-0.19	-0.12	-0.18	-0.02
Number of module instances	-0.38	-0.32	-0.40	-0.19
Size of smallest module	**0.61**	**0.69**	**0.67**	**0.73**
Size of largest module	0.26	0.34	0.19	0.35
Average module size	**0.60**	**0.68**	0.59	**0.70**
Module size standard deviation	0.23	0.29	0.13	0.29
IC-content (lowest)	0.46	0.45	**0.73**	0.47
IC-content (total)	**0.75**	**0.73**	**0.78**	0.54
Module/background-ratio	0.53	0.61	0.51	**0.63**

We conducted a simple correlation analysis to examine which properties of the TRANSCompel sequence sets and PWMs correlated best with the highest and average *nCC *scores obtained by the methods on these sets. "IC-content (lowest)" is the *information content *(IC) of the PWM with the lowest IC of the two involved in each sequence set. The information content of a PWM is inversely related to the amount of variability in the binding patterns from which the PWM is constructed [38]. PWMs with higher information content are more specific and match only sites with a high degree of similarity to the consensus motif. "IC-content (total)" is the sum of IC-contents for the two motifs (for TRANSFAC PWMs we used the PWM with the highest IC in each equivalence set to represent the motif). The three highest values are highlighted in each column. The properties that seem to correlate best with methods' performances are the minimum and average size of modules (in basepairs) and the total IC-content, which would imply that module discovery is harder for datasets containing short and degenerate modules.

**Figure 3:**
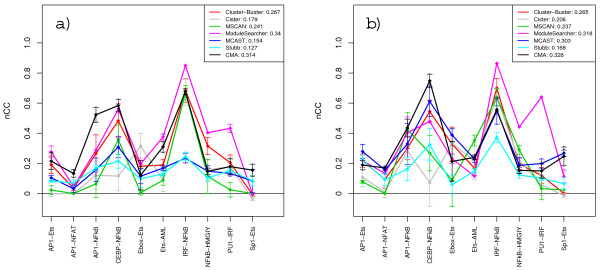
**Nucleotide-level correlation scores with 50% noise in the PWM sets**. The graphs show *nCC *scores when using TRANSFAC PWM sets (a) and custom matrices (b) with an equal proportion of decoy matrices added. Each value represents the average score over ten runs with different decoy selections.

**Figure 4:**
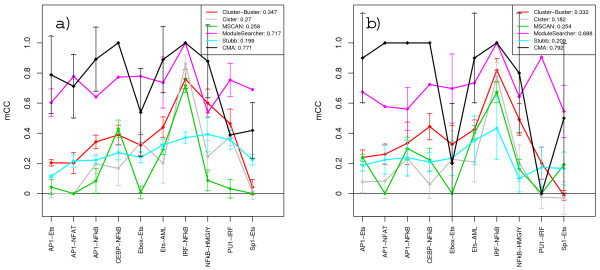
**Motif-level correlation scores with 50% noise in the PWM sets**. The graphs show *mCC *scores when using TRANSFAC PWM sets (a) and custom matrices (b) with an equal proportion of decoy matrices added. Each value represents the average score over ten runs with different decoy selections.

Figure 3 shows the results of mixing the PWM sets with an equal proportion of decoy matrices. The addition of decoy PWMs leads to a drop in score values for almost all methods. The drop is greater for the TRANSFAC PWMs, presumably because these sets contain more genuine matrices and therefore also more decoys. Contrary to expectation, some methods actually score slightly better on certain sequence sets when decoys are in use. Examples are Cister on Ets-AML and Stubb on Ebox-Ets with custom matrices. One explanation for this could be that these methods make use of decoy motifs that just happen to have a high degree of overlap with genuine modules. To examine whether the modules are predicted with the correct motifs or not, we can look at the corresponding motif-level correlation scores as shown in Figure 4. The generally high *mCC *scores obtained for IRF-NFκB support the notion that this is an easy sequence set, while the difficulty for most methods in selecting the correct motifs for CEBP-NFκB explains the higher drop seen in *nCC *for this set when decoys were added. CMA and ModuleSearcher are by far the best methods at predicting the correct composition of modules with both TRANSFAC and custom PWMs as input, although CMA does perform notably poor on two specific sequence sets. The *mCC *score for the third best method, Cluster-Buster, is less than half of that of ModuleSearcher.

**Figure 5:**
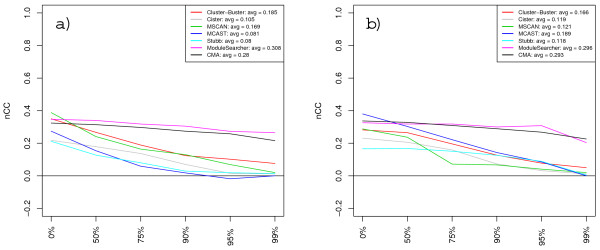
**Nucleotide-level correlation scores at different noise levels**. Plot of *nCC *scores at increasing noise levels when methods are supplied with TRANSFAC PWM sets (a) and custom matrices (b). Scores shown are averages over all sequence sets and decoy selections at each noise level. MCAST was unable to function properly with very large PWM sets and was therefore assigned a score of zero at the 99% level.

**Figure 6:**
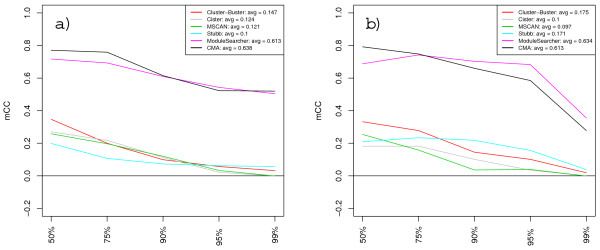
**Motif-level correlation scores at different noise levels**. Plot of *mCC *scores at increasing noise levels when methods are supplied with TRANSFAC PWM sets (a) and custom matrices (b). Scores shown are averages over all sequence sets and decoy selections at each noise level.

Figures 5 and 6 show score tendencies as increasingly more decoys are added to the PWM sets. The nucleotide-level performances of CMA and ModuleSearcher are only slightly affected by the larger amounts of decoys, whereas the scores for the other methods steadily decline. At the motif-level we clearly see a division into two groups with CMA and ModuleSearcher performing significantly better than the rest. Additional graphs detailing the effects of added noise with respect to each individual sequence set and the variations due to different decoy selections can be found at our web site.

**Figure 7:**
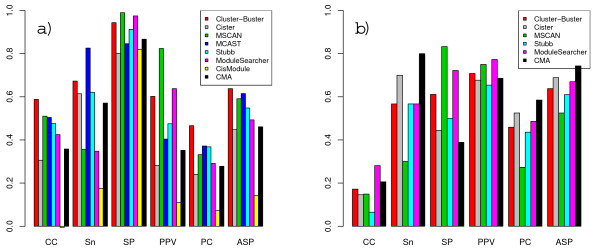
**Performances on the liver dataset**. Scores obtained on the liver dataset for different performance measures at nucleotide-level (a) and motif-level (b).

**Figure 8:**
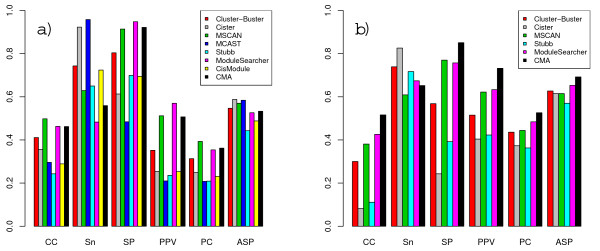
**Performances on the muscle dataset**. Scores obtained on the muscle dataset for different performance measures at nucleotide-level (a) and motif-level (b).

Results for the liver and muscle datasets are shown in Figures 7 and 8. For these datasets we supplied only four liver- and five muscle-PWMs respectively, and no decoy matrices were used. Since the modules in these datasets do not necessarily include binding sites for all of these motifs however, we could calculate motif-level scores by treating the PWMs for the missing motifs as false instances. All methods, except CisModule, did a better job on locating the modules in the liver dataset than in the TRANSCompel dataset. Cluster-Buster scored highest, but Stubb showed the largest improvement in *nCC *score. The motif-level scores, on the other hand, were not very high, which can most likely be attributed to overprediction of motifs in the case of CMA and underprediction for MSCAN. Results on the muscle dataset display the same main tendencies as the other two datasets, but for the first time, CisModule obtains an *nCC *score above zero and actually bypasses one the other methods.

## Discussion

Objective benchmarking efforts are important for providing unbiased reviews of published methods and for establishing the methodological frontier with respect to bioinformatics techniques. In this study we wanted to explore benchmarking in the context of module discovery and to investigate related design issues such as dataset construction and performance evaluation.

Benchmarking of tools for composite motif discovery is harder than benchmarking of single motif discovery tools, since the former methods are more diverse with respect to input requirements and the type of predictions they make. We have aimed at creating a simple and general framework that can be used with a wide range of methods. Nevertheless, we do not provide every kind of information that programs might ask for, and not all module discovery tools can be fairly assessed with our system.

To construct the benchmark datasets we relied on real genomic sequences containing experimentally verified modules, rather than creating synthetic datasets with fabricated and planted modules. The motivation for only using real data was to avoid introducing artificial bias related to the composition and constraints of modules. Good benchmark datasets should be diverse enough to discriminate the behaviour of different methods, when possible, and provide them with a wide range of realistic challenges. For module discovery these challenges could include discovering modules with few or many single motifs, tightly clustered or widely spaced motifs and modules with highly conserved or degenerate binding sites. Ideally, benchmark datasets should also be novel to the methods tested. Currently the amount of experimental data available is too limited to achieve all of these goals. The particular dataset we have constructed based on TRANSCompel data is novel in terms of performance testing. The modules in TRANSCompel are short, however, and to include larger modules we were forced to rely on a few well-known datasets from liver and muscle regulatory regions that have been used extensively in the past for testing and possibly for designing and developing module discovery methods. Some methods might therefore be intrinsically biased to perform well on these sets. It is conspicuous, for instance, that CisModule – which was tested with muscle data in its original publication – scored comparably well to the other methods on our muscle set, yet close to zero on both the TRANSCompel and liver datasets.

We chose the *correlation coefficient *as our main statistic for evaluating and comparing module discovery methods because it captures aspects of two of the most commonly used performance measures – *sensitivity *and *specificity *– into a single score value. However, since different statistics often favour different methods, it is prudent to consider several measures to get a better comprehension of each method's qualities. The sensitivity measure (Sn), for instance, tells us to what extent a method's predictions include the true module instances. At the nucleotide level, MCAST seems overall to be the most sensitive method among those tested here, while CMA shows high sensitivity on the TRANSCompel dataset. Yet, to achieve these high sensitivity scores the methods at the same time make a lot of false positive predictions, as can be seen from the lower *positive predictive values *(PPV). MSCAN and ModuleSearcher, on the other hand, generally have the highest nucleotide-level PPV scores, which tells us that the positive predictions made by these two programs are more trustworthy than predictions made by the other programs.

PWMs from the TRANSFAC database were used to represent both the true motifs and the decoys for the TRANSCompel dataset. A potential problem when using TRANSFAC is that many of the matrices are quite similar to each other [28]. This is partly due to some TFs being represented by several PWMs, but also because different TFs might bind to similar-looking motifs. As a result, module discovery programs can be unduly penalized for selecting an incorrect PWM at the motif level, even though the predicted PWM is very similar to the target. We have tried to remedy this situation by grouping together PWMs that correspond to the same TFs and consider these as the same motif with respect to scoring. However, there might still be other matrices in the decoy sets that can match with the annotated binding sites.

Since we are using real genomic sequences, some of the predicted modules that we label as false positives can in fact represent unannotated true positives, and so the actual performance of methods might very well be better than indicated, especially at high noise levels.

It should be noted that while the annotated length of a TF motif may vary from binding site to binding site, the length of a standard PWM is fixed, and PWMs do not always match the locations of their corresponding binding sites precisely. Perfect *nCC *scores can therefore be difficult or even impossible to obtain. The *nCC *score also drops fast if a method predicts a larger module region than what is annotated, even though the target module is correctly covered by the predicted region. This can severely penalize methods that tend to predict large module regions, especially on the TRANSCompel dataset where most modules are rather short.

Some programs can utilize additional information to strengthen confidence in predictions and improve their performance. For instance, Stubb is a sensitive method and the predictions it makes usually include the correct modules, especially when using large PWM sets; yet, its *CC*-scores are generally low because it simultaneously predicts a lot of false positives. Stubb can employ a phylogenetic footprinting [29] strategy to filter out many of these false predictions, but it requires that orthologous sequences from related species are supplied along with the regular sequences. However, in order to make the tests as comparable as possible, we have not made such additional information available to the programs in our benchmark test, unless the type of information can be expected to be readily obtained for any dataset.

Caution should thus always be taken when interpreting score values, since the reported scores might not accurately reflect the optimal capabilities of the methods. Also, we have run the programs using mostly their default parameter settings. We are fully aware that adjusting the parameters can greatly affect the performance of a program, however, selecting the most appropriate parameter values be can be tricky and running methods with default settings is probably closer to typical usage.

It is inherently difficult to conduct an assessment that is fair to all methods. Even the most minute design choice can influence the outcome if it unintentionally favours some methods over others. For instance, limiting the size of input sequences will be beneficial for most module discovery tools since it improves the signal-to-noise ratio. On the other hand, using too short sequences can disadvantage methods that require substantial amounts of data in order to derive elaborate background models. The best solution, then, is to try to balance the scales by subjecting methods to several different situations with datasets exhibiting a range of characteristics. This will make it harder still to declare a winner, since it will inevitably lead to even greater variation in the results. Then again, the purpose of benchmarks tests need not be to identify a single program that can be recommended for all needs, but rather to determine how different methods behave under different conditions, thus enabling us to select the most appropriate tool to use in specific situations.

The results from our assessment of eight published module discovery tools show that the top scoring method does vary a lot between datasets. On the TRANSCompel dataset, for instance, all methods save Stubb and CisModule score better than the others on at least one sequence set. But there is also a tendency for some methods to perform consistently better or worse across several datasets. CisModule performed poorly on most sequence sets, Cister and Stubb usually scored somewhere in the middle, while CMA, ModuleSearcher, MSCAN and Cluster-Buster were often found among the top scoring methods on each set. CMA and ModuleSearcher were clearly best at identifying the correct motif types involved in the modules, and they were also the only methods capable of coping with large and noisy PWM sets. The other PWM-reliant methods appear to be more suited for detecting modules with some prior expected composition than for discovering completely new and uncharacterized modules.

There was some variation when using custom PWMs as opposed to TRANSFAC PWM sets. The average performance over all methods on the whole TRANSCompel dataset was about the same in both cases, but there were a lot of local differences between sequence sets. The most extreme example can be seen on the Ebox-Ets sequence set where MSCAN scores highest of all with TRANSFAC matrices, yet completely fails to find any true modules with custom matrices. The average deviation in scores when using either PWM set was about 0.11 and the effect could go both ways. MCAST was the only method which almost consistently scored better with one set, namely custom matrices.

## Conclusion

While improvements can still be made to our systems, we have taken a first step towards developing a comprehensive testing workbench for composite motif discovery tools. The assessment system is based on two established datasets for module discovery plus a novel dataset we constructed from TRANSCompel module annotations. The performance of methods on our novel set is comparable to the previous two, demonstrating its utility as a benchmark set. Together these datasets challenge methods to discover modules with different characteristics and varying levels of difficulty.

Not surprisingly, trying to discover composite motifs *de novo *proves to be much more challenging than relying on PWMs as an aid to detect potential single binding sites. With large and noisy PWM sets, however, it becomes crucial to consider multiple instances of conserved motif combinations in order to identify true modules. In general, our study shows that there are still advances to be made in computational module discovery.

## Methods

### TRANSCompel dataset

Our main dataset was based on modules annotated in the TRANSCompel database [22], which is one of very few databases that contain entries for composite elements whose combinatorial binding effects have been verified through biological experiments. It comes in both a professional licensed version and a smaller public version. Our dataset was selected from TRANSCompel Professional version 9.4 which contains 421 annotated module sites from 152 different module classes. The largest modules registered in TRANSCompel are triplets (34 entries) with the remaining being pairs of binding sites (387 entries). To ensure a minimum of support for each module class, we considered only classes that had at least five annotated module sites. Unfortunately, this requirement excluded all triplets and left us with only pairs. After further discarding a few modules that were too weak to be detected with stringent PWM-thresholds, we ended up with ten sequence sets encompassing 81 module binding sites in 63 different sequences. The longest module spanned 135 bp with the average being 33 bp. The binding sequences of modules are specified in TRANSCompel by using uppercase letters to indicate bases of the constituent single motifs and lowercase letters for the intra-module background. We used the supplied references to the EMBL database [30] to obtain additional sequence bases flanking these module sites but set an upper limit of 1000 bp on the length of the sequences used. Most of the sequences were from human or mouse but also some other mammalian and a few viral sequences were included. Each sequence set was constructed around modules of one particular class made up of two single motifs from the following set of eleven: AML, AP-1, C/EBP, E-box, Ets, IRF, HMGIY, NF-AT, NF-κB, Sp1 and PU.1. The sequence sets contained between 4 and 16 sequences and the sequences themselves ranged in length from 294 to 1000 bp (average 884 bp). All sequences contained at least one module instance, but sometimes up to three, of the designated class. Some sequences also included annotated modules of other classes. This will usually not be a problem at low noise-levels, because the other modules will only interfere if the set of PWMs supplied to a program contains decoy matrices corresponding to the motifs involved in these modules. As the noise-level approaches 99%, however, this will inevitably happen because the PWM sets then include the complete TRANSFAC collection. Since we use real genomic data, there is also always a possibility that additional unknown modules are present in the sequences. Even so, for a particular sequence set, only module sites corresponding to the designated class of that set were considered true positives.

Although the TRANSCompel database itself does not provide matrix representations for the motifs involved in modules, its companion database TRANSFAC does [22]. Unfortunately, there is not a one-to-one correspondence between transcription factors and matrices in TRANSFAC, and a single factor (or family of factors that recognize the same motif) can be represented by several different PWMs. Instead of selecting just one canonical PWM to use for each motif, we collected all matrices related to a specific motif and treated the whole set as an equivalence class. Thus, a motif can be represented by either one of the PWMs in the corresponding set, and predicted binding sites in the sequences are considered to be instances of the same motif even if the binding sites are predicted by different PWMs from the equivalence set.

As an alternative to these TRANSFAC sets, we also constructed custom PWMs for the eleven motifs involved in our module classes. For each motif we extracted the corresponding annotated binding sites plus four flanking bases on each side from our sequences and used MEME [31] to align them and infer a PWM model for the motif. Constructing matrices from the same binding sites they will later on be used to detect introduces a circularity which will probably make these sites easier to find than if the PWMs had been constructed from independent sequences. This was intentional, however. Since the purpose of our study was to assess the methods' abilities to find significant *combinations *of binding sites rather than individual sites, we wanted the individual sites to be easily detectable. To verify that the annotated single binding sites in the TRANSCompel dataset were indeed detectable by our matrices, we used an algorithm from the "TFBS" package [32] to match the PWMs against the sequences. Of the 81 single binding sites in the dataset, all but ten could be detected with an 85% relative cut-off threshold. When we lowered the cut-off to 75%, all sites could be detected. Single binding sites were considered to be detected if a predicted match to the corresponding PWM overlapped with the annotated binding site. For the TRANSFAC matrices, we regarded it as sufficient if any one of the matrices in the equivalence set made a prediction that overlapped with the annotated site.

### Liver and muscle datasets

The liver dataset was based on a set of regulatory regions used as a positive training set to develop a model of liver specific regulation in the paper by Krivan and Wasserman [23]. Sequence data as well as PWM models of four TFs implied in liver specific regulation (C/EBP, HNF-1, HNF-3 and HNF-4) was downloaded from their supplementary web site [33]. After inspection of the sequence annotations, we discarded from further consideration those regulatory regions that only contained a single TFBS and also smaller annotated regions that were completely overlapped by larger regions. Furthermore, we ignored a small set of TFs that only had one binding site each in the whole dataset. This left us with regulatory regions consisting of two or more binding sites for the four TFs previously mentioned. The start position of the first TFBS and the end position of the last TFBS in each region were used as module boundaries, and the modules thus obtained varied in length from 26 to 176 bp with and average of 112 bp. Long sequences were cropped to a maximum of 1000 bp. The resulting dataset after curation consisted of 14 modules in 12 sequences with 51 binding sites for 4 different TFs. Eight of the sequences were human, two were from rat and the last two from mouse and chicken.

For the muscle dataset we selected a subset of the regulatory regions from the paper by Wasserman and Fickett [7] obtained from their web site [34]. Five motifs (Mef-2, Myf, Sp1, SRF and Tef) were reported as important in muscle regulation, and PWMs for these motifs were downloaded from the same site. We chose regions that had at least two annotated binding sites for motifs in this set and used the first and last binding site in the regions to delimit the modules. Since most of the sequences at the website were excerpts and rather short, we tried to extend them where possible by obtaining the original sequences from EMBL, though limiting the sequences to a maximum of 1000 bp as usual. The final muscle dataset used contained 24 sequences with one module in each and a total of 84 TFBS for 5 motifs. The smallest module spanned 14 bp and the longest 294 bp (average 120 bp). 10 sequences were from the mouse genome, 6 from human, 5 from rat, 2 from chicken and 1 from cow.

Further statistics on the datasets and PWMs used are summarized in Table 1 and Additional File 1.

### Running the programs

Most of the methods tested could be run directly from the input sequences and a set of PWMs. Both CMA and ModuleSearcher, however, rely on separate programs to match the PWMs against the sequences in a preprocessing step. For ModuleSearcher we used the program MotifScanner since both of these methods are part of the Toucan tools suite for regulatory sequence analysis [35]. MotifScanner was run with a third order background model based on vertebrate promoter sequences, which was also available with Toucan. CMA comes bundled with Match [36] for PWM scanning. Match utilizes two different threshold values which should be individually fitted for each specific PWM. Preconstructed cut-off profiles for TRANSFAC matrices are available for different conditions, for instance to minimize either the false positive or false negative discovery rate or to minimize the sum of these two rates. As suggested in the CMA publication, we used cut-off profiles designed to minimize the false negative discovery rate. Similar cut-off profiles for the liver, muscle and custom matrices were estimated according to the procedure described for Match [36]. For each PWM we generated 50000 random oligos by sampling from the PWM distribution. The PWM was then scored against these oligos with Match, and a cut-off threshold was chosen so that 90% of the oligos obtained a match score above this threshold. Since CMA is based on a discriminative model, it also requires a set of negative sequences along with the positive dataset. As negative data we selected 1000 bp promoter segments from 50 random housekeeping genes that were part of the default negative gene set included with the method's web service [37].

## Availability and requirements

The web service for assessing composite motif discovery tools, as well as all the results from our benchmark test, is available at .

## Abbreviations

ASP, average site performance (defined as (Sn + PPV)/2); bp, base pair; FN, false negative; FP, false positive; HMM, hidden Markov model; mCC, motif-level correlation coefficient; nCC, nucleotide-level correlation coefficient; PC, performance coefficient (defined as TP/(TP + FN + FP)); PPV, positive predictive value (defined as TP/(TP + FP)); PWM, position weight matrix; Sn, sensitivity (defined as TP/(TP + FN)); Sp, specificity. (defined as TN/(TN + FP)); TF, transcription factor; TFBS, transcription factor binding site; TN, true negative; TP, true positive.

## Authors' contributions

GKS and OA conceived of the study. All authors participated in the design of the study. KK and GKS assembled the datasets. JJ implemented the web service and ran all the tests together with KK. KK drafted the manuscript. FD was the project supervisor. All authors helped revise and approved the final manuscript.

## Supplementary Material

Additional File 1 **Dataset statistics**. This supplementary table includes information about the datasets and modules therein, the matrices used to represent the true motifs and the number of matrices in the PWM sets at various noise levels on the TRANSCompel dataset. Click here for file
